# Clinical Comparison of ^99m^Tc Exametazime and ^123^I Ioflupane SPECT in Patients with Chronic Mild Traumatic Brain Injury

**DOI:** 10.1371/journal.pone.0087009

**Published:** 2014-01-24

**Authors:** Andrew B. Newberg, Mijail Serruya, Andrew Gepty, Charles Intenzo, Todd Lewis, Daniel Amen, David S. Russell, Nancy Wintering

**Affiliations:** 1 Myrna Brind Center of Integrative Medicine, Thomas Jefferson University, Philadelphia, Pennsylvania, United States of America; 2 Department of Neurology, Thomas Jefferson University, Philadelphia, Pennsylvania, United States of America; 3 Department of Radiology, Thomas Jefferson University, Philadelphia, Pennsylvania, United States of America; 4 Magee Rehabilitation Hospital, Philadelphia, Pennsylvania, United States of America; 5 Amen Clinics, Inc., Newport Beach, California, United States of America; 6 Institute for Neurodegenerative Disorders, New Haven, Connecticut, United States of America; 7 Yale University School of Medicine, New Haven, Connecticut, United States of America; University Of Cambridge, United Kingdom

## Abstract

**Background:**

This study evaluated the clinical interpretations of single photon emission computed tomography (SPECT) using a cerebral blood flow and a dopamine transporter tracer in patients with chronic mild traumatic brain injury (TBI). The goal was to determine how these two different scan might be used and compared to each other in this patient population.

**Methods and Findings:**

Twenty-five patients with persistent symptoms after a mild TBI underwent SPECT with both ^99m^Tc exametazime to measure cerebral blood flow (CBF) and ^123^I ioflupane to measure dopamine transporter (DAT) binding. The scans were interpreted by two expert readers blinded to any case information and were assessed for abnormal findings in comparison to 10 controls for each type of scan. Qualitative CBF scores for each cortical and subcortical region along with DAT binding scores for the striatum were compared to each other across subjects and to controls. In addition, symptoms were compared to brain scan findings. TBI patients had an average of 6 brain regions with abnormal perfusion compared to controls who had an average of 2 abnormal regions (p<0.001). Patient with headaches had lower CBF in the right frontal lobe, and higher CBF in the left parietal lobe compared to patients without headaches. Lower CBF in the right temporal lobe correlated with poorer reported physical health. Higher DAT binding was associated with more depressive symptoms and overall poorer reported mental health. There was no clear association between CBF and DAT binding in these patients.

**Conclusions:**

Overall, both scans detected abnormalities in brain function, but appear to reflect different types of physiological processes associated with chronic mild TBI symptoms. Both types of scans might have distinct uses in the evaluation of chronic TBI patients depending on the clinical scenario.

## Introduction

Traumatic Brain Injury (TBI) is a major public health concern. Although the medical community has long understood the potential detriment of head injury [1], there has been a resurgence of interest regarding the importance of TBI in individuals participating in contact sports, patients with various psychological or neurological dysfunction, and the population in general. In addition, it has been observed that TBI survivors, even those with mild injury, commonly face a range of clinical symptoms affecting functional status, cognition, and mood [2]. While not everyone with a mild brain injury has lasting symptoms, those who do often present daunting problems regarding the differential diagnosis and treatment [3], [4]. Furthermore, since TBI can be heterogeneous in terms of its definition, effects, and prognosis, it is often unclear how a given TBI or multiple TBIs in the same individual will affect the brain’s physiology and how such changes may affect clinical symptoms [5], [6]. For those individuals managing patients with a history of TBI and persistent symptoms, knowledge of the underlying brain physiology may ultimately be highly beneficial for understanding the effects of the TBI and perhaps even target future therapy. Functional neuroimaging is increasingly considered an important clinical tool in the management of TBI patients [7]–[9]. This may especially be the case in patients with a history of TBI with chronic symptoms. Importantly, functional brain imaging may help remove some of the stigma associated with chronic symptoms such as headache, difficulty concentrating, or mood changes that are otherwise difficult to evaluate [10]. If a physiological basis for such symptoms can be established, patients can better understand the nature of their symptoms and hopefully be more compliant with treatment, whether it is medical, occupational, physical, or psychological. For example, post traumatic headache, which occurs in up to 70% of patients in the first year and 25% after the first year [11], is frequently debated as an entity due to a variety of factors including medicolegal influences and a pathophysiology that is not completely understood [12]. The possible pathophysiological mechanism may be related to neurogenic inflammation characterized by locally increased blood flow, plasma protein leakage from blood vessels, mast cell degranulation, and platelet aggregation [13]. This inflammation can be related to the head injury process itself or may be related to direct injury to the trigeminal afferent nerves or to the leptomeningeal or cerebrovascular structures that are innervated by trigeminal nerves [14]. The inflammatory response is likely mediated by glial cells which release a variety of cytokines that cause the release of pain modulators from trigeminal neurons ultimately resulting in headache.

Over the past two decades, a number of researchers, including our group, have focused on the utility of single photon emission computed tomography (SPECT) brain imaging to evaluate the neurophysiology of a variety of conditions and disorders. Depending on the tracer used, SPECT imaging can be useful for studying cerebral blood flow (CBF), dopamine transporter function, serotonin transporter function, as well as other processes [15]–[17]. Several studies have also shown that SPECT imaging of CBF can be helpful in TBI patients [18]. Specifically, CBF SPECT scans can demonstrate areas of impaired brain function which can help identify if trauma is present and which brain system or systems are most affected. Common findings on SPECT imaging in TBI patients include the following [9], [15], [19], [20]:

- focal decreased CBF near the focal site of injury and/or opposite side (contra coup)
- asymmetrical hypoperfusion in the prefrontal, temporal, parietal or occipital lobes
- decreased CBF in the anterior temporal poles
- decreased CBF in the contralateral cerebellar perfusion.

Early studies suggested that SPECT scans may aid in understanding a TBI patient’s symptomatology [21], [22]. More recently, some investigators have suggested that SPECT imaging could even assist clinicians in developing treatment strategies. For example, decreased prefrontal cortex perfusion, which can be associated with executive dysfunction arguably may be helped with psychostimulants [23], [24] or other interventions to enhance the function of the frontal lobes. Temporal lobe hypoperfusion has been associated with irritability and mood instability that may be improved with anticonvulsant or antidepressant medication. However, studies and case reports have reported variable success in the management of TBI patients [25], [26]. Furthermore, since different pharmacological treatments have not always demonstrated a benefit in this population, future studies are required to better assess how well certain clinical and imaging findings can be used to direct therapy.

SPECT may be more useful than other structural imaging techniques such as magnetic resonance imaging (MRI) or x-ray computed tomography (CT) in TBI patients [27]. TBI patients often report symptoms such as headaches, memory loss, concentration difficulties, perceptual sensitivities, dizziness, or emotional liability, even when CT and/or MRI scans demonstrate no clear abnormalities [28], [29], [30]. Such patients may be labeled as somatic or malingering, especially when there are no significant neuroimaging abnormalities present. However, researchers investigating the differences between functional and structural imaging techniques have found SPECT to be more sensitive for detecting abnormalities in patients with varying degrees of TBI [31]. Thus, it is possible that patients with TBI will demonstrate functional abnormalities on SPECT imaging that may relate to their persistent clinical symptoms.

In addition to alterations in CBF, a growing amount of evidence implicates damage to neurotransmitter systems, such as the dopaminergic nervous system, in association with TBI [32]. For example, after experimental TBI, regional increases in dopamine levels at acute time points after acceleration–deceleration brain injury have been observed [33]. Interestingly, cortical tissue dopamine levels are actually depressed for up to 2 weeks post brain injury [34]. Increases in human cerebrospinal fluid (CSF) dopamine and its metabolites post-TBI appear to depend on both gender and genetic variations in the dopamine transporter (DAT). Increased dopamine metabolism may represent a compensatory response to increased tissue dopamine levels or may be due to additional direct effects of TBI on the regulation of the dopamine system. The consequences of such changes might be beneficial in that dopamine neurotransmission is restored after TBI or could potentially be deleterious due to dopamine-induced oxidative stress.

Another line of evidence regarding the dopaminergic effect of TBI is that dopamine receptor agonists often benefit patients via the promotion of central dopaminergic transmission [23], [24]. This could be a sign that dopamine release is suppressed after injury, that dopamine uptake by the DAT is increased, or some combination of the two. Alternatively, dopamine activity may remain normal after injury, but this level of dopamine activity is not adequate in the face of the injury-induced disruptions. Given that a few studies have demonstrated beneficial effects with dopamine antagonists, it should also be acknowledged that TBI results in a complex series of injuries to a wide range of different brain structures. It is important to acknowledge that any systemic treatment for TBIwithout a well-defined window for therapy could be both beneficial and/or detrimental to the recovery process. For this reason, understanding the effect of TBI on dopamine transmission is crucial for developing a proper therapeutic plan and for promoting optimal recovery.

The current study was designed to evaluate for the first time CBF and dopamine transporter binding in the same patient with TBI and chronic symptoms. Two reviewers read the scans together using a visual scoring system based upon common clinical approaches to evaluating these scans so that this data could be more easily utilized in current clinical practice. Scan findings that were considered mild, moderate, or severe, could then be provided a numerical value to assist in a more quantitative analysis of the data. This approach was similar to previous comparison studies performed by our group for functional neuroimaging studies [35], [36]. Thus, the overall purpose of this study was to assess the ability of ^99m^Tc Exametazime (Ceretec™; GE Healthcare) SPECT to detect changes in CBF and compare those changes to DAT binding evaluated with ^123^I Ioflupane (DaTscan™; GE Healthcare) SPECT in the same TBI patients with chronic symptoms. These scan findings were then compared to clinical symptoms and neuropsychological test measures of mood, health, and cognitive function. The goal is to help further develop the use of SPECT imaging in TBI patients presenting with chronic symptoms by determining how each of these two SPECT tracers may help clinicians better evaluate such patients.

## Materials and Methods

### Ethics Statements

This research was approved by the Thomas Jefferson University Institutional Review Board. Written informed consent, approved by the Thomas Jefferson University Institutional Review Board, was received from all patients who participated in the study. The clinical Investigation was conducted according to the principles expressed in the Declaration of Helsinki.

### Subjects Selection

Twenty-five patients (14M/11F; mean age 51±16) with one or more prior traumatic brain injuries with chronic symptoms (see demographics in Table 1) were recruited. Subjects were recruited primarily from the Neurology Department at Thomas Jefferson University and also from the Concussion Clinic at Magee Rehabilitation Hospital both in Philadelphia. All subjects were evaluated with both ^99m^Tc Exametazime and ^123^I Ioflupane SPECT. The scans from these subjects were also compared to 10 ^99m^Tc Exametazime SPECT scans from control subjects with no history of head injury (5M/5F; mean age 54±6) and 10 ^123^I Ioflupane SPECT scans from another group of control subjects also with no history of head injury (5M/5F; mean age 57±14). ^123^I Ioflupane SPECT scans were obtained for the age and gender matched healthy control subjects from the Parkinson Progression Marker Initiative database (www.ppmi-info.org) and subjected to the identical analysis. All TBI patients had a history of at least one prior head injury over 6 months prior to the scan with continued symptoms that included memory loss or other cognitive problems (N = 20), headaches (N = 9), emotional problems such as anxiety or depression symptoms (N = 11), or other symptoms (see Table 1 for patient demographics and TBI history). Most subjects were currently employed or were students such that their TBI symptoms did not limit their capacity to function. Subjects did not have other neurological disorders or meet criteria for chronic traumatic encephalitis.

**Table 1:** Demographic and clinical information for TBI patients.

Subject #	Gender	AGE(at study)	Total Numberof TBIs	Cause of TBI	Concurrent CNS Conditions
1	M	76	1	Sports	Memory loss, migraine headaches,
2	M	61	2	Sports	Memory loss, attention problems, anxiety, depression
3	M	27	3	Blast	Memory loss, attention deficits, anxiety, depression
4	F	56	2	Sports	Memory loss, headaches
5	F	69	1	Sports	Depression
6	F	27	2	Sports	Headaches
7	M	64	4	MVA	Depression, anxiety
8	M	18	1	Sports	Loss of concentration, apathy
9	F	34	1	Sports	Severe vertigo, memory loss, attention/concentration problems
10	M	58	4	Sports	Memory loss, headaches
11	F	61	3	MVA	Headaches, dizziness, memory loss, sleep disturbances
12	F	50	1	MVA	Memory loss, attention/visual problems
13	M	58	1	Accident	Vertigo, sleep problems, memory loss
14	M	36	1	Accident	Memory loss, attention deficits
15	F	39	5	Accident	Memory loss, attention deficits, language problems, anxiety, depression
16	F	25	3	Sports	Memory loss, attention deficits, depression, anxiety, apathy
17	F	65	2		Memory loss, anxiety
18	M	68	1	MVA	Memory loss, tremors, agitation, disinhibition
19	M	61	8	MVA	Headaches, memory loss, language problems, attention deficits
20	M	62	2	MVA	Severe headaches, depression
21	F	58	1	MVA	Visual problems, headaches, tinnitus, memory loss
22	F	70	3	MVA	Memory loss, anxiety
23	M	46	1	MVA	Memory loss, attention deficits, depression
24	M	42	3	MVA	Memory loss, balance problems, falls
25	M	52	1	MVA	Visual problems, memory loss, headaches, vestibular problems

MVA = motor vehicle accident; Accident = other type of accidental trauma.

All subjects were evaluated with several neuropsychological questionnaires to assess health and emotional status including the Profile of Mood Scale (POMS) which measures six identifiable mood or affective states: Tension-Anxiety, Depression-Dejection, Anger-Hostility, Fatigue-Inertia, and Confusion-Bewilderment [37], the Spielberger State-Trait Anxiety Inventory (STAI) to measure anxiety [38], the Beck Depression Inventory (BDI) to measure depressive symptoms [39], and the Short Form-12 (SF-12) for general health [40] which is also subsequently divided into the Physical Component Score (PCS) and Mental Component Score (MCS).

### SPECT Acquisition

All scans were performed at the Thomas Jefferson University Department of Nuclear Medicine according to their standard clinical protocols. Approximately 10 minutes prior to injection, an intravenous cannula (IV) was placed in one arm. For CBF scans, each subject was injected with their eyes open and ears unoccluded in a dimly lit room with limited ambient environmental stimuli. Subjects received the recommended ^99m^Tc Exametazime dosage in the package insert of 370–740 MBq (10–20 mCi) intravenously. Imaging was performed on a Philips Forte dual headed SPECT scanner with ultrahigh resolution low energy collimators approximately 30 minutes after injection and acquired over 30 minutes. Images were reconstructed in the axial, sagittal, and coronal planes using filtered back projection and Chang’s attenuation correction. Within one month of the CBF SPECT scan, subjects underwent dopamine transporter SPECT imaging. For this scan, patients received 16 drops of Lugol’s solution for thyroid blocking approximately 30 minutes prior to injection. Ten minutes prior to injection, an IV canula was placed in one arm. Each subject received 111 to 185 MBq (3 to 5 mCi) of ^123^I Ioflupane intravenously as per the package insert. They underwent scanning approximately 3 hours after injection for 45 minutes.

### Image Interpretation

All SPECT scans were interpreted by two board certified nuclear medicine physicians with extensive experience interpreting brain SPECT scans. The reviewers interpreted all the scans together by consensus in a randomized order for both the CBF and DAT SPECT scans. The reviewers were blinded to any clinical information. For the CBF scans, the assessment was performed to qualitatively rate the cerebral blood flow in the four major cortical brain regions – the frontal, temporal, parietal, and occipital lobes, as well as the anterior and posterior cingulate, caudate, basal ganglia, thalamus and cerebellum. With the left and right hemispheres included, there was a total of 18 brain regions evaluated. Each structure was rated for CBF in both the left and right hemisphere. We used a previously described approach for interpreting functional imaging scans [35], [36] such that the CBF of each anatomic structure on the SPECT scan was given a score: 4 = normal activity; 3 = mildly decreased activity; 2 = moderately decreased, 1 = severely decreased; and 0 = no activity. DAT SPECT images were assessed visually using a similar semiquantitative score ranging from 4 = normal binding; 3 = mildly decreased binding; 2 = moderately decreased binding, 1 = severely decreased binding; and 0 = no binding. Four regions were evaluated, the left and right caudate, and the left and right putamen.

### Data Analysis

Initially, descriptive statistics were used regarding the number and extent of abnormal activity on the CBF and DAT binding scans from the TBI patients and controls. A linear regression model was used to compare the neuropsychological test scores to CBF scores in relevant regions for the entire cohort of TBI patients. For DAT binding scans, since there was a limited dynamic range of values given only 4 relevant brain regions, a t-test was used to compare those patients with highest and lowest quartiles of the neuropsychological test scores to the overall striatal DAT binding. Significant findings were cross validated with non-parametric tests (Wilcoxon Two Sample Test for comparison of groups and Spearman rank analysis for correlations) to account for possible skewness of the data. Finally, the activity for the CBF scans and DAT binding were compared to each other particularly in the basal ganglia, but also between the frontal regions on CBF scans and the basal ganglia on the DAT scans. Where indicated, correction for multiple comparisons was made using the False Discovery Rate method [41].

## Results

**Figure 1:**
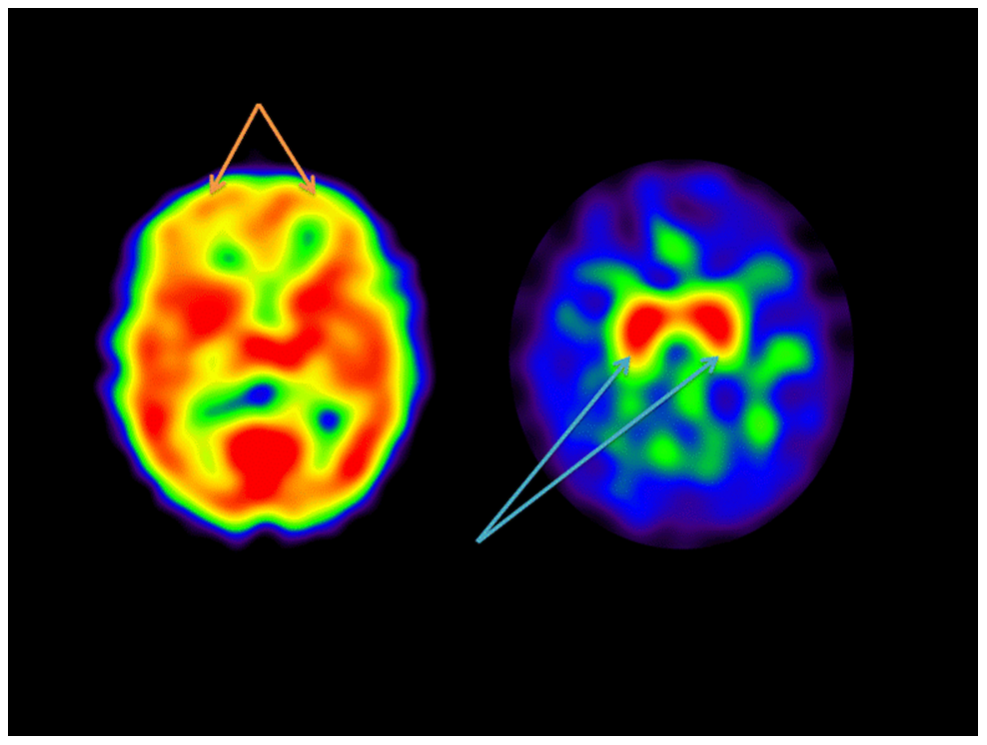
^99m^Tc Exametazime and ^123^I Ioflupane SPECT studies in a 61 year old male subject with multiple prior TBIs presenting with headache and memory loss. Note the markedly decreased CBF primarily in the frontal lobes bilaterally (yellow arrows). However, this same patient had robust DAT binding in the striatum (blue arrows) with no significant abnormalities.

**Figure 2:**
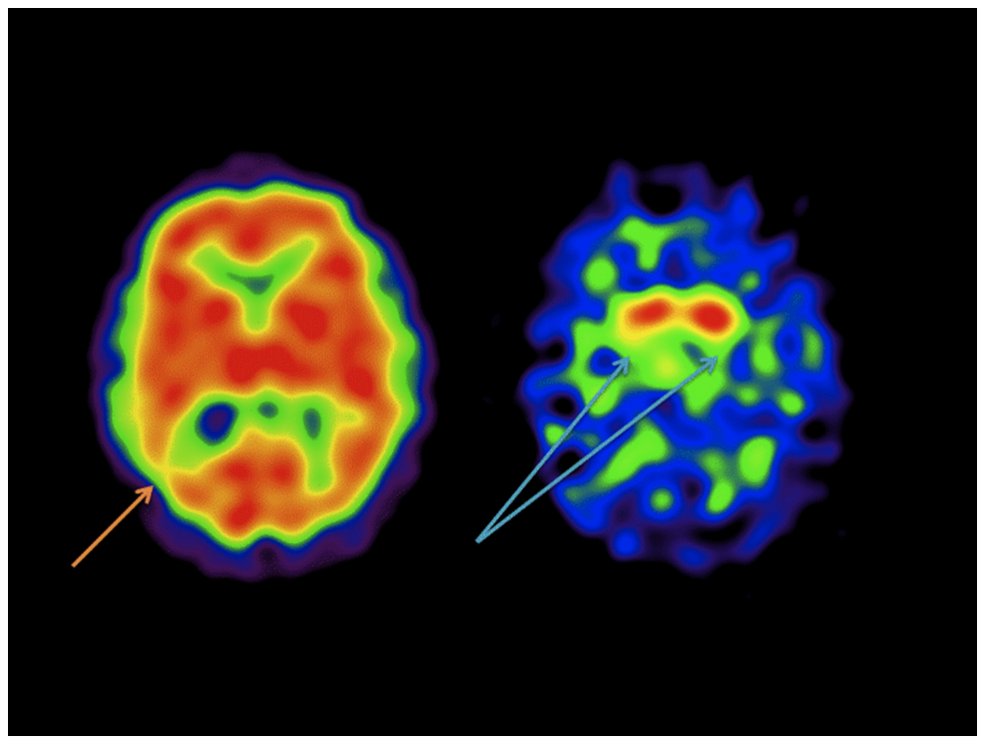
^99m^Tc Exametazime and ^123^I Ioflupane SPECT studies in a 58 year old male subject with a history of a single TBI with clinically presenting with problems with memory, sleep, and vertigo and no clear parkinsonian features. The CBF shows only a mild decrease in the right temporo-parietal region. However, DAT binding is markedly abnormal with preserved uptake primarily in the caudate (R>L) and minimal uptake in the putamen bilaterally.

Patients with a history of TBI and persistent symptoms generally had one or more abnormal perfusion findings on their brain scans (see Figures 1 and 2). In fact, TBI patients had an average of 6 brain regions with abnormal perfusion compared to controls who had an average of 2 abnormal regions out of 18 regions evaluated (see Table 2). In controls, all decreases in perfusion were mild whereas in the TBI group, 10 of 25 patients had regions graded as moderate or severely decreased. Given the sample size, a sensitivity and specificity between the groups could not be performed, however, the number of abnormal regions was significantly higher in the TBI group compared to the control group (p<0.001). On the ^123^I Ioflupane SPECT, 72% of TBI patients had abnormal DAT binding compared to 20% of controls (p = 0.01). However, the findings in the controls were typically mild and restricted to a single putamen, which may have even been related to artifact, whereas the TBI patients were more likely to have involvement of the caudate or both putamen. Interestingly, the TBI patients in this cohort had the abnormalities more in the left striatum with significantly lower (by Wilcoxon Two Sample Test) binding in the left caudate and left putamen compared to controls (left caudate mean of 3.6±0.6 in TBI and 4.0±0.0 in controls, p = 0.02; left putamen mean of 3.3±0.7 in TBI and 3.8±0.3 in controls, p = 0.01).

**Table 2:** General evaluation results of the 99mTc Exametazime and 123I Ioflupane SPECT scans (note that several additional regions were evaluated but not listed).

		99mTc Exametazime Results								123I Ioflupane SPECT Results				
TBISubject	R Front	L Front	R Par	L Par	R Temp	L Temp	R Striat	L Striat	R Thal	L Thal	R Caud	L Caud	R Put	L Put
**1**	ABN	ABN	N	N	N	N	N	ABN	ABN	ABN	N	ABN	ABN	ABN
**2**	N	N	ABN	N	N	N	N	ABN	N	N	N	N	N	ABN
**3**	N	N	ABN	N	N	ABN	N	ABN	N	N	N	N	N	N
**4**	N	ABN	N	N	N	ABN	N	ABN	N	ABN	ABN	N	ABN	N
**5**	N	ABN	N	ABN	N	ABN	ABN	ABN	N	ABN	N	N	N	N
**6**	ABN	ABN	N	N	N	ABN	N	ABN	N	ABN	N	N	N	ABN
**7**	ABN	ABN	N	N	N	N	N	ABN	N	ABN	N	ABN	N	ABN
**8**	ABN	N	N	ABN	N	ABN	N	ABN	N	N	N	ABN	N	ABN
**9**	ABN	ABN	ABN	N	ABN	N	ABN	ABN	N	N	N	N	N	N
**10**	N	N	ABN	N	ABN	N	N	N	ABN	N	N	ABN	N	N
**11**	ABN	N	ABN	N	N	N	N	ABN	N	ABN	N	N	N	ABN
**12**	N	ABN	N	N	ABN	N	ABN	N	N	N	N	ABN	N	ABN
**13**	N	N	ABN	ABN	ABN	N	N	N	N	N	ABN	N	ABN	ABN
**14**	ABN	N	ABN	N	N	N	ABN	N	N	N	N	N	ABN	N
**15**	N	ABN	N	ABN	N	ABN	N	ABN	N	ABN	N	N	ABN	ABN
**16**	N	ABN	ABN	N	ABN	N	N	ABN	N	ABN	N	ABN	N	ABN
**17**	N	ABN	N	N	N	ABN	N	N	N	N	ABN	N	ABN	N
**18**	N	ABN	N	ABN	N	ABN	ABN	N	N	N	N	N	N	N
**19**	ABN	ABN	N	N	ABN	ABN	N	N	ABN	ABN	N	ABN	N	ABN
**20**	ABN	ABN	N	N	ABN	ABN	N	ABN	N	ABN	N	N	N	N
**21**	ABN	ABN	N	N	ABN	ABN	ABN	ABN	ABN	ABN	N	ABN	N	ABN
**22**	N	N	N	N	N	N	N	N	ABN	ABN	N	N	N	N
**23**	N	ABN	N	ABN	ABN	ABN	N	ABN	N	N	N	N	N	ABN
**24**	N	ABN	N	ABN	N	N	ABN	ABN	N	ABN	N	N	N	N
**25**	ABN	ABN	N	N	N	N	ABN	ABN	ABN	ABN	N	ABN	N	ABN
**Control**														
**1**	ABN	N	N	N	N	N	N	N	N	N	N	N	ABN	N
**2**	N	ABN	N	N	N	N	ABN	ABN	N	N	N	N	N	N
**3**	ABN	ABN	ABN	ABN	N	N	N	N	N	N	N	N	N	N
**4**	N	N	N	N	N	N	ABN	N	ABN	N	N	N	N	N
**5**	N	N	N	N	N	ABN	N	N	N	N	N	N	N	N
**6**	N	N	N	N	N	N	N	N	N	N	N	N	N	N
**7**	N	N	N	N	N	N	N	ABN	N	ABN	N	N	ABN	N
**8**	N	ABN	N	N	N	N	N	N	N	N	N	N	N	N
**9**	N	N	ABN	ABN	N	ABN	N	N	N	N	N	N	N	N
**10**	N	ABN	N	N	N	ABN	N	N	N	N	N	N	N	N

N = Normal; ABN = abnormal; R Front = Right Frontal; L Front = Left Frontal; R Par = Right Parietal; L Par = Left Parietal; R Temp = Right Temporal; L Temp = Left Temporal; R Striat = Right Striatum; L Striat = Left Striatum; R Thal = Right Thalamus; L Thal = Left Thalamus; R Caud = Right Caudate; L Caud = Left Caudate; R Put = Right Putamen; L Put = Left Putamen.

When CBF was compared to DAT binding, there were no clear associations between findings. Abnormal CBF in the caudate or basal ganglia did not appear to predict or correspond to abnormal DAT binding. Even asymmetries were not comparable with 10 patients having asymmetric CBF and 11 patients having asymmetric DAT binding, but concordance in only 2 patients.

We performed a limited set of comparisons between clinical symptoms, neuropsychological test scores, and CBF or DAT binding based upon expected associations observed in prior studies. There were some interesting findings in the current cohort. For patients reporting headaches, there were significant findings compared to those patients without headaches. Patients with headaches had lower right frontal (3.8±0.4 without vs. 3.1±0.7 with headaches, p = 0.02) and increased left parietal CBF (3.4±0.6 without vs. 4.0±0.0 with headaches, p = 0.03 by Wilcoxon Two Sample Test). Please note that the findings for the right frontal lobe and left parietal lobe survived correction for multiple comparisons. Interestingly, there was no clear association between those reporting cognitive impairment or the type of impairment (i.e. loss of concentration, loss of memory, etc.) and either CBF or DAT binding. However, this lack of a finding might be due in part to so many patients reporting symptoms (only 3 patients reported no cognitive symptoms).

With regard to specific neuropsychological measures, there were also several interesting findings related to CBF and DAT binding. For the SF-12 PCS, there was a significant correlation between low CBF in the left frontal lobe and reports of lower physical health (r = 0.50, p = 0.01 corrected). Similarly, there was a significant correlation (Spearman rank correlation) between low CBF in the right temporal lobe and reports of lower physical health (r = 0.54, p = 0.005 corrected). The implication is that those patients with worse perceived physical health have lower CBF in these regions. Total CBF score for all regions correlated negatively with fatigue (r = 0.44, p = 0.03) but did not correlate with any other measures on the POMS. Furthermore, there were no significant findings between the MCS and CBF values.

For DAT binding, those patients in the lowest quartile for depression compared to the highest quartile had significantly higher binding in the total striatum (3.3±0.7 and 3.7±0.2 respectively, p = 0.01 by Wilcoxon Two Sample Test). This is consistent with our prior depression data which demonstrated increased DAT binding in patients with depression and also in controls with depressive symptoms [42], [43]. For the SF-12 MCS, the results for those with scores in the lowest quartile versus the highest quartile showed DAT binding of 3.8±0.2 and 3.3±0.6 respectively (p = 0.02 by Wilcoxon Two Sample Test). Finally, there was higher DAT binding in patients with the highest quartile scores on the Spielberger trait anxiety scale versus those who were in the lowest quartile (3.8±0.1 versus 3.2±0.7, p = 0.02 by Wilcoxon Two Sample Test).

## Discussion

The overall purpose of this study was to compare CBF using ^99m^Tc Exametazime SPECT and DAT binding using ^123^I Ioflupane SPECT in patients with persistent symptoms associated with a history of mild TBI. As described below, several studies have evaluated CBF or DAT binding alone in similar cohorts of patients, but not together [44], [45], [46]. For example, prior research studies have explored the relationship between CBF findings on SPECT imaging and TBI both in terms of diagnosis and clinical symptoms.

Some early studies compared the value of CBF SPECT imaging to anatomical findings on MRI or CT in TBI patients. Ichise et al. [19] studied 29 patients with chronic symptoms after either mild or major head injury. SPECT imaging detected abnormalities in 66% patients whereas MRI detected abnormalities in 45% and CT in 34% of patients. The SPECT scans also correlated better with tests of memory, attention and executive function than did structural imaging. A study of 43 patients with persistent post-concussive symptoms demonstrated abnormal SPECT scans in 53% of patients while MRI was read as abnormal in 9% and CT scan in only 5% of patients [44]. Although the SPECT scan results appeared to be more sensitive in detecting cerebral abnormalities in these patients, current neuropsychiatric symptoms did not seem to correlate with the SPECT scan findings. A more recent study of patients with acute mild TBI demonstrated CT abnormalities in 34% of patients while SPECT showed abnormal CBF in 63% corroborating earlier findings [45]. As in our study, the most common abnormality on many of these previous studies was hypoperfusion in the frontal lobes. We also found on average 6 regional abnormalities in CBF in the TBI group suggesting a more widespread pathophysiological process similar to other studies [20].

In the current study, we found a number of correlations between CBF and various clinical or neuropsychological parameters. For example, patients reporting headaches as part of their post-TBI symptoms had significant differences in the CBF in their right frontal lobe, and left parietal lobe. Such findings, which may reflect diffuse axonal injury and inflammation, are also consistent with several other studies of headache and pain syndromes [47], [48], [49]. An early study reported decreased cerebral blood flow is associated with common migraines suggesting that migraine attacks occur in connection with exacerbations of preexisting changes of neuronal activities, cerebral perfusion and metabolism [50]. In addition, in our study, low CBF in the left frontal lobe and right temporal lobe correlated with poorer reported physical health. The finding of decreased left frontal CBF is consistent with the well-known association of left dorsolateral prefrontal cortical dysfunction and depressed mood. Just as ischemic stroke, arteriovenous malformations, and neoplastic mass lesions in this area are often associated with depressed mood, this study suggests that TBI induces a ‘functional lesion’ of this area altering self-perception of health in a manner consistent with depressive thinking (anhedonia and despair) [51], [52], [53].

Other studies have evaluated the correlation between SPECT CBF findings and clinical findings as well as prognosis. For example, one study evaluated the results of neuropsychological tests in 12 alert, responsive patients aged 18–26 years, who were 2–13 months after head injury and found that global CBF was significantly decreased in patients with head injury relative to age-matched controls [54]. In that study, 3 of 4 patients with a well-localized injury had focally decreased CBF over the affected region. Also in that cohort, 2 of 3 patients with diffuse injury who underwent repeat studies 5–14 weeks after their initial study had improvement on both their psychological tests and CBF. These results suggested that the chronic sequelae of head injury include decreased CBF, presumably reflecting decreased cerebral metabolism related to diffuse axonal injury or continued inflammatory response, which correlates with the neuropsychological impairment. Our current study supports this overall hypothesis given the generally reduced CBF in patients with chronic symptoms related to prior TBI.

A more thorough evaluation of the correlation between SPECT findings and prognosis was found in a study by Jacobs et al. which prospectively evaluated 67 mild-to-moderate brain injured patients [55]. Each patient had a clinical evaluation and SPECT scan within four weeks of the initial injury and three months after the first scan. This study reported that of the 33 patients who showed no significant abnormalities on their initial SPECT scan, 97% experienced resolution in their clinical symptoms within three months. However, of the 34 patients with abnormalities on their initial SPECT scans, 59% continued to experience significant clinical symptoms three months later. The authors reported a positive predictive value of an abnormal initial scan of only 59%, but if the 12 month follow up scan was also abnormal, the sensitivity for the repeat SPECT was 95%. These authors suggested that negative initial SPECT studies are a reliable predictor of a favorable clinical outcome. The results showed that SPECT alterations correlated well with the severity of the trauma and a negative initial SPECT study was a reliable predictor of a favorable clinical outcome. The authors suggested that in cases with a positive initial SPECT scan, a follow-up consisting of a combination of SPECT and clinical evaluation is necessary especially in patients suffering from post-conclusive symptoms. Our current study would corroborate the notion that positive SPECT scans are associated with chronic symptoms in TBI patients.

Another prospective study evaluated 136 patients with mild head injury who underwent initial SPECT imaging within 4 weeks after the trauma and again up to 12 months post injury [56]. That study revealed that clinical normalization occurred earlier than normalization of CBF. However, at 12 months post injury, the investigators observed considerable improvement in the specificity and positive predictive value of SPECT (85% and 83%, respectively). Furthermore, persistent lesions on the SPECT scan were correlated with severity and to localization in the frontal cortex. The authors suggested that a normal 99mTc-HMPAO SPECT scan early in the post TBI course may be a reliable tool for predicting improvement. As with our study, patients with persistent symptoms frequently have a variety of CBF abnormalities even a year or more after TBI.

A study by Bonne et al. [57] studied 28 clinically symptomatic male subjects with mild TBI and twenty matched controls with neuropsychological testing and brain SPECT imaging. Similar to our study, mild TBI patients demonstrated regions of hypoperfusion in the frontal lobes, temporal lobes, and sub-cortical structures which was generally concordant with neuropsychological localization. The results from these CBF SPECT studies, in addition to the results from the current study support using SPECT to help assess the diagnosis, prognosis, and treatment of TBI patients.

In addition to alterations in CBF, a growing amount of evidence implicates damage to the dopaminergic nervous system in association with TBI. Additional evidence of dopamine system dysfunction after TBI is based upon data showing altered DAT binding after TBI. One study of only 10 subjects used single photon emission computed tomography (SPECT) with beta-CIT to show that striatal DAT binding was decreased in patients up to 4–5 months after severe TBI, even in cases where there was no evidence of structural striatal injury [46]. Patients with TBI also had significantly lower D2 post-synaptic receptor binding as measured by IBZM SPECT. However, the DAT deficit was more marked than the D2 receptor loss. The findings suggested that nigrostriatal dysfunction could be detected using SPECT following TBI despite relative structural preservation of the striatum. However, there still have been no studies that explored the comparison between dopamine function and cerebral blood flow as measured by SPECT.

The current study provides unique data by being able to directly compare CBF and DAT binding in the same TBI patients. Our SPECT results show that both CBF and DAT binding are abnormal in TBI patients with chronic symptoms. Furthermore, both CBF and DAT abnormalities appear to correlate with clinical symptoms. However, there does not seem to be a good correlation between CBF and DAT SPECT scan findings.

There are several limitations with regard to interpreting the findings in this study. One limitation is that the SPECT scans were all interpreted together by two expert reviewers. Thus, this study does not provide information on inter-rater or intra-rater reliability for the scans. However, the purpose of this study was to determine how these scans might be utilized in the traditional clinical setting in TBI patients so we sought to interpret the scans under optimal conditions. Given the potential importance of utilizing these scans for the clinical evaluation of chronic TBI patients suggested by the current data, future studies with larger numbers of subjects will be needed to better assess how well these scans can be evaluated by different readers under more complex conditions. However, such a study can potentially yield data to support the broader use of SPECT imaging in this population. In addition, it will be important to perform additional quantitative analyses in the future. Again though, since quantitative analysis with tools such as region of interest analysis or statistical parametric mapping is not typically used in the clinical setting, they were not performed as part of the current study. Another limitation for SPECT’s use in TBI is that typically no pre-trauma SPECT study is available for comparison. Thus, it is often not possible to determine the date of trauma with functional neuroimaging. Even remote trauma from childhood can present with neuroimaging findings similar to those seen in more recent trauma.

## Conclusion

Overall, this study provides the first data on CBF and DAT binding in the same patients with a history of TBI and chronic symptoms. The results demonstrate that such TBI patients frequently have a number of perfusion and DAT binding abnormalities. Such abnormalities appear to correlate with specific symptoms or clinical measures. But, interestingly, the scans do not appear to correlate well with each other. The implication of this last finding is that the scans appear to provide insight into two separate pathophysiological processes associated with TBI symptoms. Thus, both scans might ultimately have utility in the setting of patients with TBI and persistent symptoms.
